# Fecal Microbiomes Distinguish Patients With Autoimmune Hepatitis From Healthy Individuals

**DOI:** 10.3389/fcimb.2020.00342

**Published:** 2020-08-03

**Authors:** Jiamin Lou, Yan Jiang, Benchen Rao, Ang Li, Suying Ding, Hang Yan, Heqi Zhou, Zhenguo Liu, Qingmiao Shi, Guangying Cui, Zujiang Yu, Zhigang Ren

**Affiliations:** ^1^Department of Infectious Diseases, The First Affiliated Hospital of Zhengzhou University, Zhengzhou, China; ^2^Gene Hospital of Henan Province, The First Affiliated Hospital of Zhengzhou University, Zhengzhou, China; ^3^Precision Medicine Center, The First Affiliated Hospital of Zhengzhou University, Zhengzhou, China; ^4^Department of Neurology, The First Affiliated Hospital of Zhengzhou University, Zhengzhou, China; ^5^Health Management Center, The First Affiliated Hospital of Zhengzhou University, Zhengzhou, China

**Keywords:** autoimmune hepatitis, diagnosis, fecal microbiome, microbial biomarkers, operational taxonomic unit

## Abstract

**Objective:** The intestinal microbiome is associated with various autoimmune diseases. Regional difference is the main influencing factor of intestinal microbial difference. This study aimed to identify the differences in fecal microbiome between autoimmune hepatitis (AIH) patients and healthy controls (HCs) in Central China, and to validate the efficacy of fecal microbiome as a diagnostic tool for AIH.

**Design:** We collected 115 fecal samples from AIH patients (*N* = 37) and HCs (*N* = 78) in Central China and performed gene sequencing. Fecal microbiomes were characterized and microbial markers for AIH were identified.

**Results:** Fecal microbial diversity showed a downward trend in AIH compared with HCs. Fecal microbial communities significantly differed between both groups. At the phylum level, *Verrucomicrobi*a abundance was significantly increased, while *Lentisphaerae* and *Synergistetes* were significantly decreased in the AIH patients vs. the HCs. Compared to the HCs, 15 genera, including *Veillonella, Faecalibacterium*, and *Akkermansia*, were enriched, while 19 genera, such as *Pseudobutyrivibrio, Lachnospira*, and *Ruminococcaceae*, were decreased in the AIH patients. Ten genera, including *Veillonella, Faecalibacterium*, and *Akkermansia*, predominated in the AIH patients. Five microbial biomarkers were deemed optimal diagnostic tools for AIH. The probability of disease was significantly increased in AIH group vs. HCs, achieving 83.25% value of area under the curve.

**Conclusion:** We present the characteristics of AIH patients in Central China for the first time. Five microbial biomarkers, including *Lachnospiraceae, Veillonella, Bacteroides, Roseburia*, and *Ruminococcaceae*, achieved a high potential distinguishing AIH patients from HCs.

## Introduction

The human intestinal mucosa is extremely vulnerable to changes in the surrounding environment. Its microbial residents comprise a unique system that imperceptibly affects host immunity and metabolism (Sommer et al., 2017). Recently, the alterations in the intestinal flora characterized by perturbations in microbial composition and function were associated with autoimmune diseases such as rheumatoid arthritis (RA) (Alpizar Rodriguez et al., 2019), autoimmune liver disease (AILD) (Wei et al., 2019), multiple sclerosis (MS) (Jangi et al., 2016), and type 1 diabetes (T1D) (Vatanen et al., 2018). Increasing attention has been given to fecal microbiota as intestinal microecology plays important roles in the diagnosis and pathogenesis of numerous diseases (Sun et al., 2018).

Autoimmune hepatitis (AIH) is a chronic, non-specific liver inflammation mediated by the immune system. Epidemiological statistics show that the incidence of AIH in China is up to 20/100,000, and the average annual incidence rate of white people in northern Europe is 1.07~ 1.9/100000 (Lohse et al., 2015). Its etiology is correlated with several genetic and environmental factors but has not yet been fully elucidated (Wei et al., 2019). AIH is globally distributed and occurs in children and adults. It is characterized by etiological and clinical heterogeneity and is distinct from other hypothetical autoimmune liver diseases such as primary bile cirrhosis (PBC) and primary sclerosing cholangitis (PSC). However, it shares certain features in common with these conditions (Wang et al., 2016). Rapid and timely diagnosis is important because of the high mortality rate of untreated AIH. Long-term AIH is associated with an increased risk of cirrhosis and/or hepatocellular carcinoma (HCC). Diagnostic criteria have been established and verified for AIH. Nevertheless, there are certain confounders including autoantibody variability (such as autoantibody-negative AIH), drug-induced AIH, similarities among AIH, PBC, and PSC, overlap syndrome (OS), and post-transplant AIH (Anand et al., 2018).

There are strong correlations between the intestinal microbiome and various chronic liver diseases. Previous research attempted to establish whether there was a distinction between patients with AIH and healthy controls (HCs) in terms of their intestinal microbial communities (Li et al., 2018). Mouse model-based experiments demonstrated the involvement of intestinal microbiomes in AIH diagnosis and pathogenesis (Yuksel et al., 2015; Li et al., 2017). In a recent study, 91 patients with AIH and 98 matched HCs underwent 16S rRNA gene sequencing. The former had lower α-diversity and distinct overall microbial compositions compared to the latter (Wei et al., 2019). However, regional difference is the main influencing factor of intestinal microbial difference (He et al., 2018). Herein, we aimed to identify the differences in fecal microbiome between AIH patients and HCs in Central China, and to validate the efficacy of fecal microbiome as a diagnostic tool for AIH.

## Materials and Methods

### Participant Inclusion and Exclusion Criteria

The study was designed and performed in accordance with PRoBE (prospective specimen collection and retrospective blinded evaluation), the Helsinki Declaration, and the Rules of Good Clinical Practice (Ren et al., 2019). Before commencing the experiments, ethical clearance was sought from the First Affiliated Hospital of Zhengzhou University (No. 2017-XY-002). Written informed consent was secured for each enrolled participant.

All fecal samples originated from newly diagnosed AIH patients admitted to the outpatient department of the First Affiliated Hospital of Zhengzhou University between 2018 and 2019. A diagnosis of AIH was confirmed if the patient conformed to the following criteria: (1) 1999 International AIH Group (IAIHG) score ≥ 10, or (2) 2008 IAIHG simplified AIH score ≥ 6, or (3) characteristic AIH histology, and (4) diagnosed as AIH for the first time. Newly diagnosed patients with AIH were excluded for the following reasons: (1) diagnosis of primary biliary cholangitis (PBC), (2) diagnosis of overlap syndrome (OS), (3) antibiotic consumption within the past 2 wks, (4) diagnosis of non-alcoholic steatohepatitis (NASH), viral hepatitis, alcoholic liver disease (ALD), or drug-induced liver injury (DILI) (Balitzer et al., 2017; Sebode et al., 2018), (5) had been treated with steroids or UDCA.

Thirty-seven newly diagnosed patients with AIH and 78 age-, gender-, and body mass index (BMI)-matched healthy controls (HCs) from the physical examination department of the First Affiliated Hospital of Zhengzhou University were enrolled in the study. Fecal samples from the enrolled participants were prospectively collected and subjected to 16S rRNA Miseq sequencing.

### Human Fecal Sample Collection and DNA Extraction

Each participant provided a fresh stool sample. Routine fecal testing was performed to assess stool consistency. The samples were divided into five 200-mg pieces and immediately stored at −80°C. A QIAamp Fast DNA Stool Mini Kit (Qiagen, Hilden, Germany) was used to perform the DNA extraction (Tang et al., 2018).

### PCR Amplification and MiSeq Sequencing

The forward primers 5′-ACTCCTACGGGAGGCAGCA-3′ and the reverse primer 5′-GGACTACHVGGGTWTCTAAT-3′ targeting the hypervariable V3–V5 region (338F/806R) of the 16S rRNA gene was used in the PCR amplification of the extracted DNA. PCR amplification was performed in a 20-μL reaction system consisting of 4 μL of 5 × Fastpfu buffer, 2 μL of 2.5 mM dNTPs, 0.4 μL of forward primer (5 μM), 0.4 μL of reverse primer (5 μM), 0.4 μL of TransStart Fastpfu DNA polymerase (TransGen Biotech, Beijing, China), and 10 ng of template DNA. PCR was conducted in an ABI GeneAmp® 9700 (Thermo Fisher Scientific, Waltham, MA, USA) using the following program: 95°C for 2 min; 30 cycles of 95°C for 30 s, 55°C for 30 s, 72°C for 30 s, and a final extension at 72°C for 5 min (Ren et al., 2019). The PCR products were detected on a 2% (w/v) agarose gel and the bands were extracted and purified with AxyPrep^TM^ DNA gel (Axygen Scientific, Waltham, MA, USA) and a PCR Cleanup System (Promega, Madison, WI, USA). The purified PCR products were mixed and DNA libraries were constructed following the manufacturer's instructions. Sequencing was performed on an Illumina MiSeq Platform by Shanghai Mobio Biomedical Technology Co. Ltd., Shanghai, China (Ren et al., 2013). The raw Illumina read data were deposited in the European Nucleotide Archive Database of the European Bioinformatics Institute under accession number PRJNA556801.

### Sequence Data Processing

Before the barcodes and primers were removed, the filtered reads were assigned to various samples based on the specific barcodes. The paired-end sequenced reads of each library were overlapped with the default parameters using FLASH v. 1.2.10 (Magoc and Salzberg, 2011). The overlapped reads generated by FLASH were subjected to quality control to filter out mismatches in the barcode/primer region, ambiguous bases, and reads with >5 in the overlap region. The reads were de-multiplexed and assigned to various samples based on the barcodes. Chimeric sequences were detected and removed with UCHIME v. 4.2.40 (Edgar et al., 2011). The Broad Institute 16S “gold standard” database served as a reference (microbiome util-r20110519 version; http://drive5.com/uchime/gold.fa) to match the operational taxonomic units (OTUs).

### Operational Taxonomic Unit (OTU) Clustering and Taxonomy Annotation

After randomly selecting equal numbers of reads from all samples, the OTUs were binned with the UPARSE pipeline (Edgar, 2013) as follows: (1) Abundant sequences and singletons were removed. (2) Unique sequences were binned into the OTUs with the “usearch-cluster_otus” command. (3) The selected sequences were aligned against the OTU sequences with the “usearch-usearch_global-id 0.97” command. (4) The identity threshold was set to 0.97. (5) The OTU composition table was created (Lu et al., 2019).

The gross OTUs were counted at each taxonomic level (phylum, class, order, family, and genus). The results were presented in a statistical table listing the OTU sequence numbers of each sample.

### Bacterial Diversity and Taxonomic Analysis

Bacterial community diversity was assessed by the Shannon and Simpson indices and calculated in the “vegan” package of R. Chao and Ace estimators were used to describe bacterial community richness. Rarefaction curves were plotted to compare microbial community richness among samples and validate their sequencing data. Venn diagrams were plotted to identify the common and unique OTUs in multiple samples and reveal OTU similarity and overlap. Dominant species heatmaps were plotted with Heatmap Builder. Microbial community barplots were generated by species composition analysis (Ren et al., 2019).

A principal coordinate analysis (PCoA) was conducted with R (http://www.R-project.org/) to disclose the microbiome spaces between samples. PCoA is functionally similar to non-metric multidimensional scaling (NMDS) analysis (Ren et al., 2014). The phyloseq package was used to calculate the weighted and unweighted unifrac distances. The former were calculated in phyloseq using the command “Unifrac (X1, weighted = T, normalized = T, fast = T).” For the latter, the parameter was “weighted = F.” X1 is a composite of a sequence table and a phylogenetic tree. A custom R program function provided by EBML (http://enterotype.embl.de/enterotypes.html#dm) was used to calculate the Jensen-Shannon distance. The Spearman coefficient distance was calculated with “as.dist (1-cor (dat), method = “spearman”).” The output data comprise the OTU composition table (Ren et al., 2017).

A phylogenetic tree was plotted as follows: (1) The sequences were aligned with MUSLE. (2) Fast Tree MP was used to calculate the unrooted phylogenetic tree with a generalized time-reversible (gtr) model. (3) A custom perl script furnished by Microbes Online (reroot.pl, www.microbesonline.org/programmers.html) was used to re-root the phylogenetic tree.

Bacterial taxonomic analyses were performed at the phylum, class, order, family, and genus levels and comparisons were made between the AIH and HCs groups via a Wilcoxon rank-sum test. A linear discriminant analysis (LDA) was conducted using the linear discriminant analysis effect size (LEfSe) method (http://huttenhower.sph.harvard.edu/galaxy/root?tool_id=lefse_upload) to characterize the fecal microbiomes. The LDA was combined with a non-parametric Kruskal-Wallis rank-sum test (*P* < 0.05) and the Wilcoxon rank-sum test to screen for key biomarkers (community members). The LDA score of log_10_ = 2 was set as the cutoff value.

### Gene Function Prediction

Phylogenetic Investigation of Communities by Reconstruction of Unobserved States (PICRUSt) predicts the metabolic functions of bacterial flora and 16S rRNA gene sequences in Kyoto Encyclopedia of Genes and Genomes (KEGG), Clusters of Orthologous Groups (COG), and Rfam. The core of the KEGG database is a biological metabolic pathway analysis database (KEGG PATHWAY Database, http://www.genome.jp/kegg/pathway.html). Its metabolic pathway categories include Metabolism, Genetic Information Processing, Environmental Information Processing, Cellular Processes, Organismal Systems, and Human Diseases. Each metabolic pathway classification is subdivided into multiple grades. The second level comprises 45 metabolic pathway subfunctions, the third level corresponds to a metabolic pathway map, and the fourth level contains explanatory information for each KO (KEGG orthologous group) in the metabolic pathway.

PICRUSt predicts the metabolic functions of bacteria and ancient bacteria by comparing the 16S rRNA gene sequencing data against a reference database of microbial genomes with known metabolic functions.

The annotation information corresponding to each functional spectrum database per sample and the abundance matrix for the predicted functional groups may be obtained from the prediction results of PICRUSt.

Relative differences in 16S rRNA gene copy number among species were considered during the prediction process. The original species abundance data were corrected to enhance prediction accuracy and reliability (Wang et al., 2018).

### OTU Biomarker Identification and Probability of Disease (POD) Construction

A random forest model (R 3.4.1; random forest 4.6–12 package) was used to select significantly different OTUs in each sample group. The generalization error was estimated by 10× cross-validation. The OTU frequency profile was generated by mapping reads from the AIH and HC groups onto these represented sequences (Fouhy et al., 2019). The cross-validation error curve was plotted after the 10× cross-validation. The cutoff point was that with the lowest cross-validation error. The sum of the minimum error and the SD at the corresponding point were defined as the cutoff value. All sets of OTU markers with errors below the cutoff value (≤30) were listed. The optimal set was that with the fewest OTUs. The minimal OTU combination revealing differences between both groups with the highest accuracy was identified. Subsequent analysis such as receiver operating characteristic curve (ROC) was then performed. Statistical significance was determined with a Wilcoxon rank-sum test (*P* < 0.05) (Deschasaxux et al., 2018).

The POD index was defined as the ratio of the number of randomly generated decision trees predicting samples as “AIH” to that of healthy controls. To evaluate the diagnostic efficacies of the selected biomarkers, the ROC was plotted and the area under curve (AUC) was calculated using pROC (R 3.8.1) (Tilg et al., 2018).

### Statistical Analysis

SPSS v. 20.0 (IBM Corp., Armonk, NY, USA) was used to process the data. Statistical significance of the differences between both groups was calculated. A Wilcoxon rank-sum test was conducted to compare the continuous variables between groups. Fisher's exact test was used to compare categorical variables between groups. Spearman's rank test was used for the correlation analysis.

## Results

### Characteristics of the Participants

One hundred and fifteen stool samples were prospectively collected from 37 newly diagnosed patients with AIH and 78 age-, gender-, and BMI-matched HCs after a strict selection and exclusion process. Clinical characteristics of the AIH and control groups are shown in Table 1. Most of the AIH patients were middle-aged and elderly women. Clinical characteristics were matched between the AIH and HCs groups (*P* > 0.05). Liver function indices such as alanine aminotransferase (ALT), aspartate aminotransferase (AST), γ-glutamyltransferase (GGT), and total bilirubin (TB) were higher in the AIH patients than the HCs group (*P* < 0.001). Compared to the HCs group, serum albumin (ALB) was significantly lower in the AIH patients (*P* < 0.001).

**Table 1:** Clinical characteristics.

**Characteristics**	**AIH (*n* = 37)**	**HC (*n* = 78)**	***P-*value**
Age, years, median (min–max)	50 (25–72)	49.5 (36-65)	0.469
Gender, Female, *n* (%)	34 (91.89%)	67 (85.9%)	0.358
BMI, kg/m^2^, median (min–max)	21.89 (18.47–29.34)	22.04 (18.31–29.34)	0.962
**HEPATIC FUNCTION, MEDIAN (MIN–MAX)**			
ALT, U/L	160 (47–553)	16 (7–38)	0.000-
AST, U/L	95 (40–407)	20 (11–33)	0.000
AKP, U/L	70 (40–263)	69 (32–157)	0.296
GGT, U/L	90 (54–321)	17 (7–49)	0.000
TB, umol/L	20 (6–71)	11.305 (3.8–18.12)	0.000
ALB, g/L	36 (29–54)	47.35 (39.6–53.2)	0.000
**IMMUNOGLOBULIN, MEDIAN (MIN–MAX)**			
IgG, g/L	21.3 (9.82–44)		
IgM, g/L	1.5 (0.51–4.2)		
IgA, g/L	1.8 (0.6–8.63)		
**AUTOANTIBODY**, **±**, **+%**			
ANA	33/4; 89.19%		
ASMA	5/32; 13.51%		
SLA/LP	9/28; 24.32%		

*Continuous variables were compared using Wilcoxon rank sum test between both groups. Fisher's exact test compared categorical variables. Statistical analyses were performed using SPSS version 20.0 for Windows (SPSS Inc., Chicago, IL)*.

*BMI, body mass index; AIH, autoimmune hepatitis; ALT, alanine aminotransferase; AST, aspartate aminotransferase; AKP, alkaline phosphatase; GGT, gamma-glutamyltransferase; TB, total bilirubin; ALB, albumin*.

### Data Quality and Intestinal Microbial Diversity in AIH Patients

The rarefaction analysis disclosed that OTU richness in each group approached saturation. Microbial richness was higher in the AIH (*n* = 37) than the HCs (*n* = 78) group (Figure 9A). Similar results were obtained for the Shannon-Wiener and Rank-Abundance curves (Figures 9B,C).

**Figure 1:**
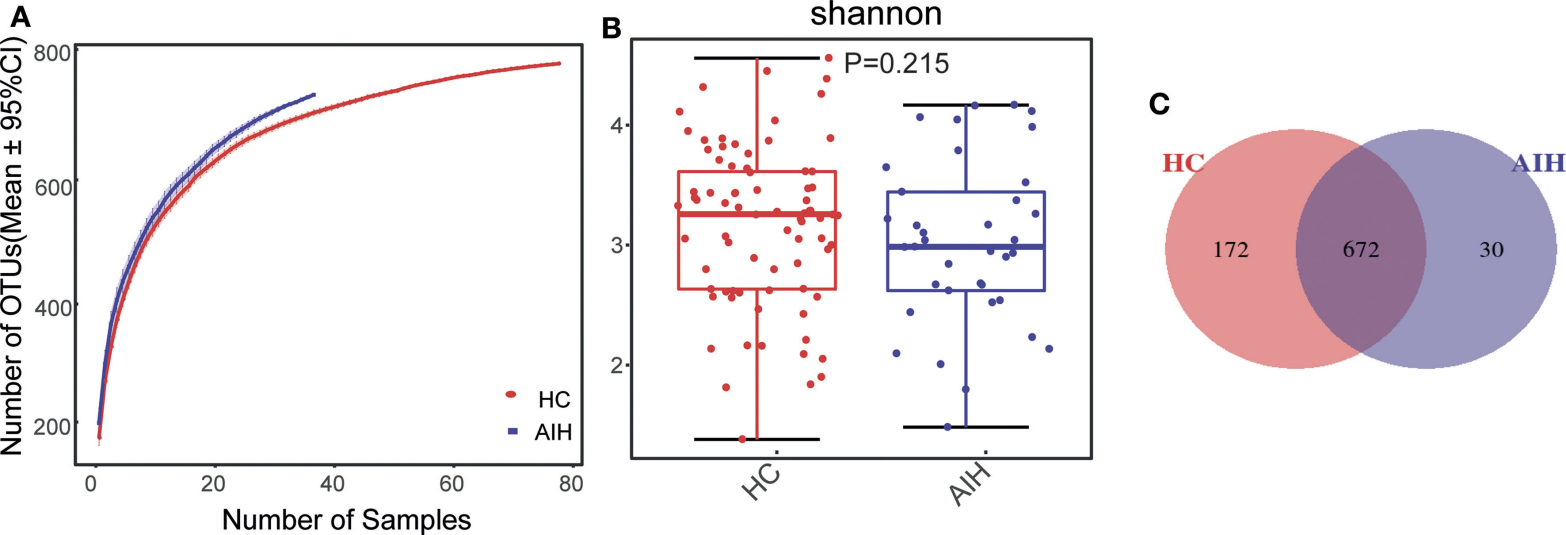
Relative reduction of fecal microbial diversity in patients with AIH (*N* = 37) and HCs (*N* = 78). **(A)** Specaccum species accumulation curves for number of samples and estimated richness. Estimated OTU richness approached saturation in all samples. Fecal microbial diversity estimated by Shannon index **(B)** was significantly decreased in patients with AIH (blue) compared with HCs (red). **(C)** Venn diagram displaying overlaps between groups showed that 672 of the 874 OTUs were shared between the AIH (blue) and HCs (red) groups. Thirty of the 874 OTUs were unique to AIH. AIH, autoimmune hepatitis; HCs, healthy controls; OTUs, operational taxonomic units. Significant differences by *P < 0.05; **P < 0.01, ***P < 0.001.

The Specaccum species accumulation curves (Figure 1A) revealed that OTU richness approached saturation in all samples. The Shannon index (Figure 1B) of AIH group was lower than that of HCs group, but the difference was not statistically significant (*P* = 0.215). Fecal microbiome diversity showed a downward trend in AIH compared with HCs.

The Venn diagram showed that 672 of the 874 OTUs were common to both groups (Figure 1C) while 30 of them were unique to AIH. Notably, compared with HCs group, there were 172 OTUs lost in AIH group, implying the microbial differences between AIH and HCs group.

### Differences in Fecal Microbiome Between AIH and Healthy Individuals

**Figure 2:**
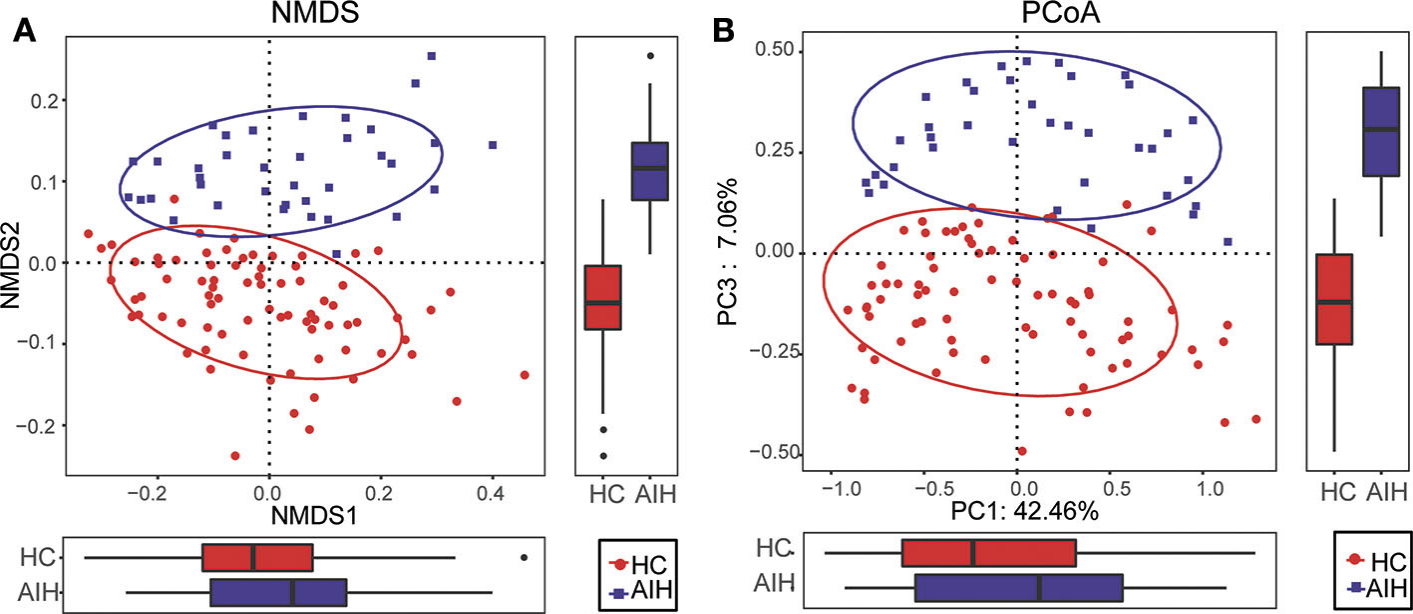
Comparisons of β-diversity in fecal microbiomes of AIH (*N* = 37) and HCs (*N* = 78). **(A)** β-diversity was calculated by NMDS analysis of unweighted UniFrac. Samples of AIH (blue) and HCs (red) groups were distinctly separated in the direction of the NMDS2 axis which means that individuals with AIH were substantially different from healthy individuals. **(B)** PCoA of unweighted UniFrac PC1-3 showed that the samples of the AIH (blue) and HCs (red) groups were distinctly separated in the direction of the PC3 axis which means that the overall fecal microbiota compositions were markedly different between AIH and HCs. Each symbol represents a sample (blue, AIH; red, HCs). Variance explained by the PCs is indicated by parentheses on the axes. AIH, autoimmune hepatitis; HCs, healthy controls; NMDS, non-metric multidimensional scaling; PCoA, principal coordinate analysis.

The β-diversity was determined by NMDS analysis of unweighted UniFracIn and described the microbiome space between both groups. Samples from the AIH (blue) and HCs (red) groups separated in the direction of the NMDS2 axis. Therefore, the fecal microbial communities in patients with AIH were markedly distinct from those of the HCs (Figure 2A).

Similar results were obtained for the PCoA of unweighted UniFrac PC1-3 (Figure 2B). Samples from the AIH (blue) and HCs (red) groups separated in the direction of the PC3 axis. This finding confirmed that the fecal microbial communities were substantially different between the AIH patients and the HCs.

**Figure 3:**
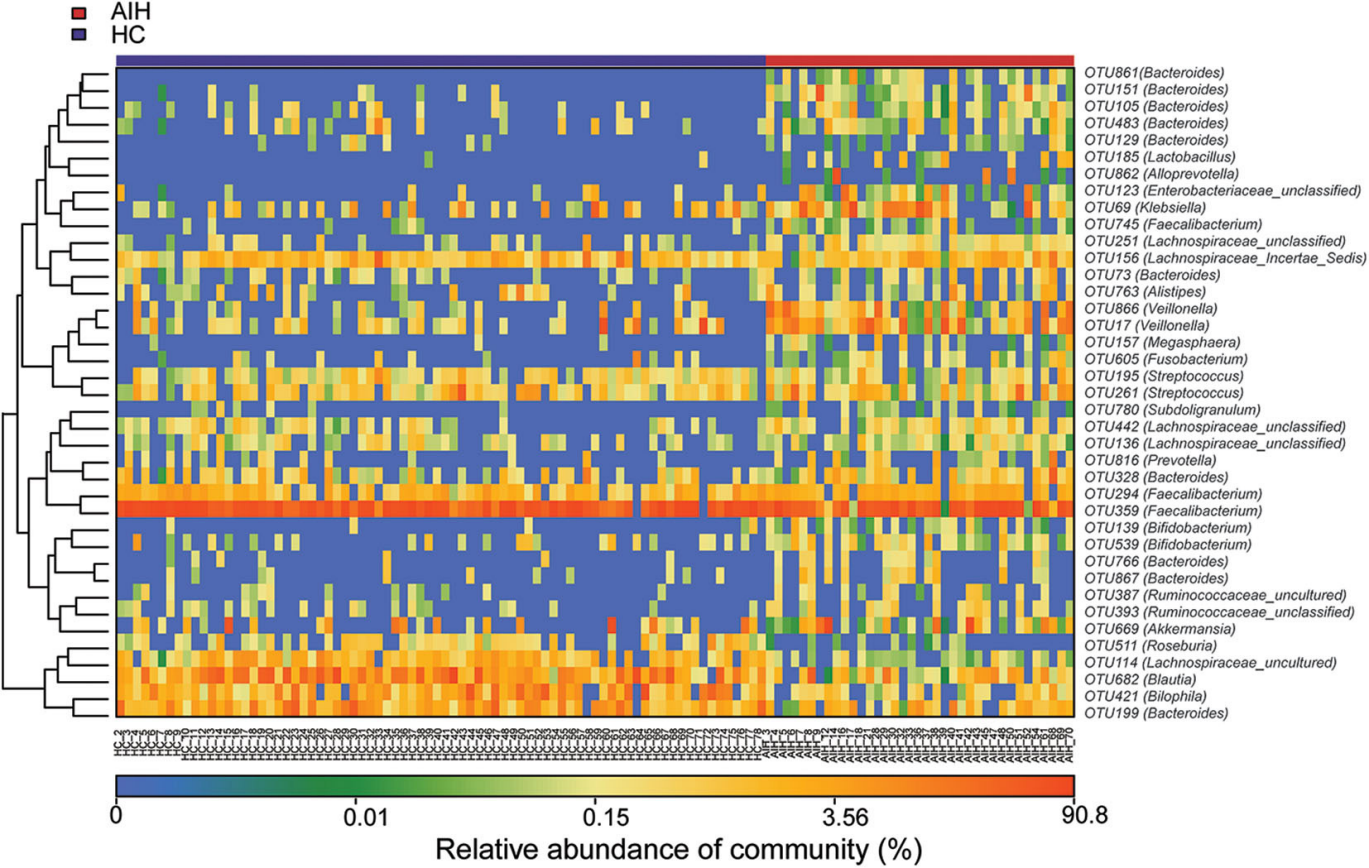
Heatmaps for relative abundances of differential OTUs between AIH (*N* = 37) and HCs (*N* = 78). For each sample, the columns show relative abundance data for differential OTUs on the right. The relative abundance of each OTU was used to plot the heatmap (blue, low abundance; red, high abundance). Group data are shown above the plot: HCs, left, blue line; AIH patients, right, red line. Each row represents one OTU. AIH, autoimmune hepatitis; HCs, healthy controls; OTUs, operational taxonomic units.

The microbial community heatmap showed that 34 OTUs including *Enterobacteriaceae, Veillonella, Ruminococcaceae_uncultured, Roseburia*, and *Bacteroides* were enriched in the fecal microbial communities of the AIH patients relative to those of the HCs. In contrast, five OTUs including *Bacteroides, Bilophila, Blautia*, and *Lachnospiraceae_uncultured* were drastically depleted in the fecal microbiomes of patients with AIH compared with those of the HCs (Figure 3). In order to show the divergence more intuitively, we further use histogram (Figure 10) to exhibit that there are significant differences in the abundance of five OTUs including OTU387 (*Ruminococcaceae_uncultured*), OTU151 (*Bacteroides*), OTU678 (*Roseburia*), OTU866 (*Veillonella*), and OTU114 (*Lachnospiraceae_uncultured*) between AIH and HCs groups (*P* < 0.05).

### Composition and Comparison of Fecal Microbiomes in AIH and HCs

Relative microbial abundances of each fecal sample at the phylum, class, order, family, and genus levels were calculated and plotted on basis of their OTU annotations. To compare the fecal microbial communities at each taxonomic level between the AIH patients and the HCs, significant differences in microbial composition between groups were analyzed by a Wilcoxon rank-sum test. False discovery rates (FDR) and *q* were calculated for *P* (Cohen et al., 2019).

**Figure 4:**
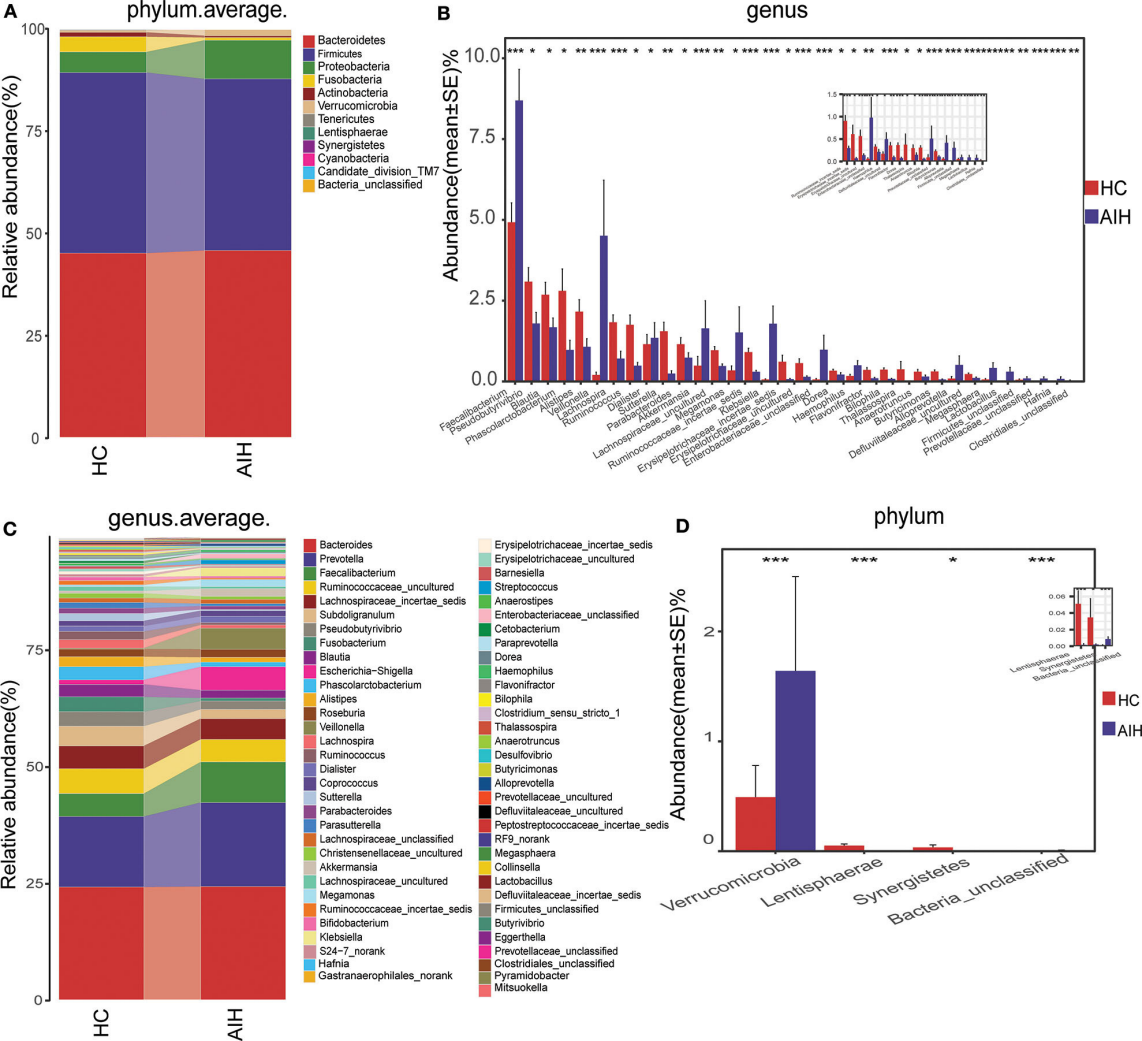
Composition and comparison of fecal microbiomes in AIH (*N* = 37) and HCs (*N* = 78). Composition of fecal microbiota at the **(A)** phylum and **(B)** genus levels in AIH versus. HCs. Comparison of fecal microbiota at the **(C)** phylum and **(D)** genus levels between groups. Significant differences in the abundance of predominant genera between AIH (blue) and HCs (red). The average abundance of each bacterium is depicted as mean ± SE. *P*-values were calculated by a Wilcoxon rank-sum test and shown in Supplementary Data S1, S2. *P < 0.05; **P < 0.01; ***P < 0.001; AIH, autoimmune hepatitis; HCs, healthy controls.

Microbial community barplots for the AIH and HCs groups reflected relative microbial abundances at the phylum and genus levels for each sample (Figures 11A,B). At the phylum level, the cumulative average proportion of *Bacteroidetes, Firmicutes*, and *Proteobacteria* for AIH and HCs was 90% and there were no significant changes between both groups. Phyla ranking fourth in the AIH and HCs groups were *Verrucomicrobia* and *Fusobacteria*, respectively (Figure 4A). At the genus level, *Bacteroides, Prevotella, Faecalibacterium, Ruminococcaceae*_uncultured, *Lachnospiraceae*_incertae_sedis, *Subdoligranulum, Pseudobutyrivibrio*, and *Fusobacterium* accounted on average for > 60% in both groups (Figure 4B). Twelve families including *Verrucomicrobia, Lactobacillaceae, Leptotrichiaceae, Enterobacteriaceae*, and *Veillonellaceae* were significantly higher, while 16 families including *Alcaligenaceae, Victivallaceae, Erysipelotrichaceae, Acidaminococcaceae*, and *Lachnospiraceae* were significantly lower in the AIH patients.

The abundance of phylum *Verrucomicrobia* (*P* = 0.0005; *q* = 0.002) in the patients with AIH was significantly higher than that in the HCs group. In contrast, *Lentisphaerae* (*P* = 0.0007; *q* = 0.002) and *Synergistetes* (*P* = 0.0275; *q* = 0.0579) were significantly lower in the AIH than the HCs group (Figure 4C).

The abundances of 15 genera such as *Veillonella, Faecalibacterium, Klebsiella, Akkermansia, Enterobacteriaceae_unclassified*, and *Megasphaera* were significantly higher in the AIH than the HCs group. The abundances of 19 genera including *Pseudobutyrivibrio, Blautia, Lachnospira, Erysipelotrichaceae_incertae_sedis, Ruminococcaceae_incertae_sedis, Phascolarctobacterium*, and *Alistipes* were significantly lower in the AIH than the HCs group (Figure 4D).

At the phylum and genus levels, certain fecal microbial communities such as *Verrucomicrobia*, Lentisphaerae, *Synergistetes, Faecalibacterium*, and *Veillonella* were significantly different between the AIH and HCs groups (*P* < 0.05) (Supplementary Data S1, S2). Moreover, significant differences were observed between the AIH and HCs groups at the microbial class, order and family levels (Figures 12A–C) (Supplementary Data S3–S5) (*P* < 0.05).

### Phylogenetic Characteristics of The Fecal Microbial Communities in AIH Patients

**Figure 5:**
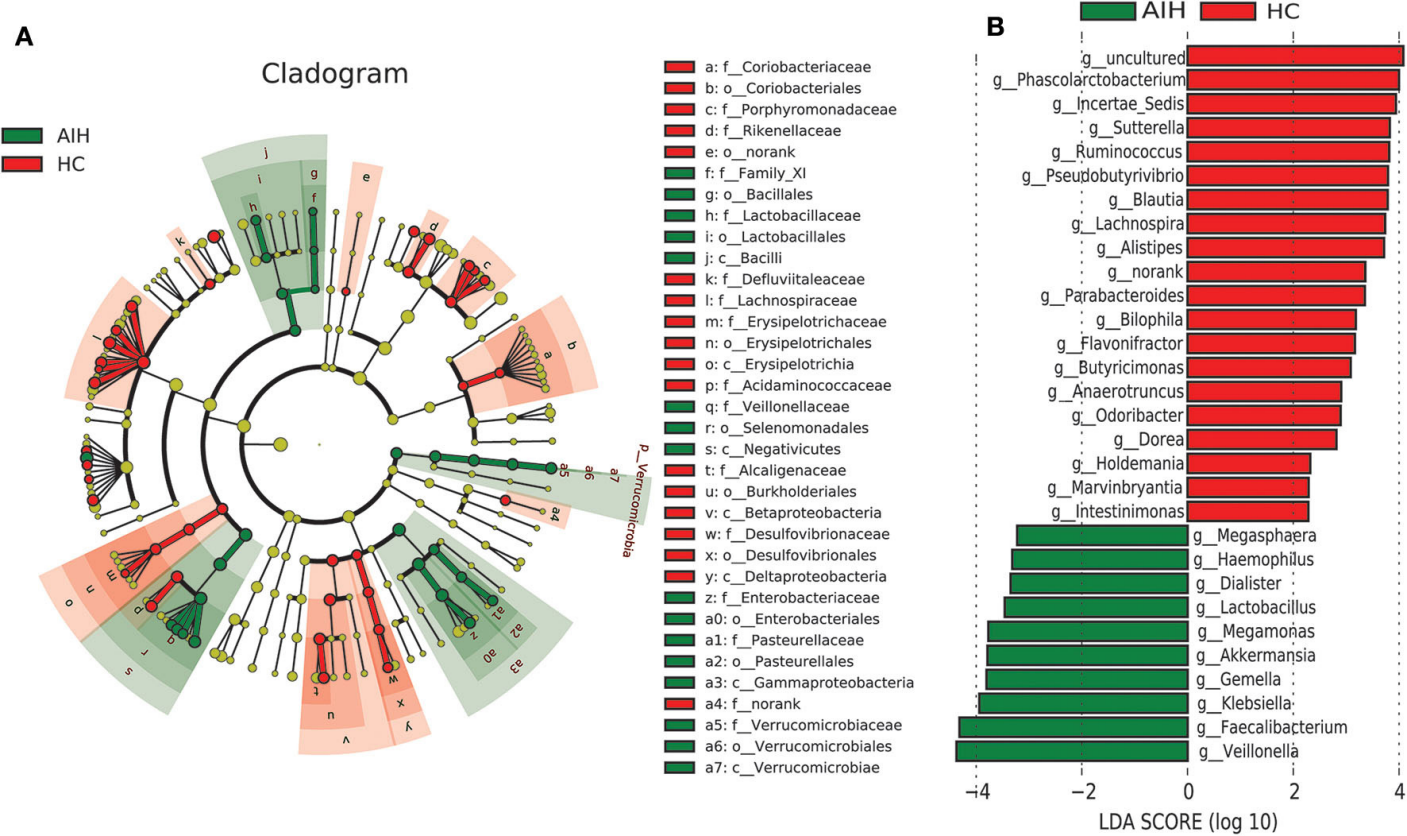
LEfSe and LDA analyses based on OTU characterizations of microbiota in AIH (*N* = 37) and HCs (*N* = 78). **(A)** Cladogram generated by the LEfSe method indicating phylogenetic distribution of fecal microbiomes associated with AIH patients and HCs. Each filled circle represents one phylotype. Phylum and class are indicated by name on the cladogram. Order, family, and genus are listed on the right panel. Circle size is proportional to phylotype abundance. By default, it is arranged outward from phylum to genus. Red circles in the branches represent microbial communities playing pivotal roles in AIH. Green circles represent microbial groups playing important roles in HC. Yellow circles represent microbial groups of little significance in either group. Default LDA > 2 and *P* < 0.05 indicate different species and higher abundance in one group than the other. **(B)** Histogram of LDA scores calculated for selected taxa showing significant difference in microbe type and abundance between AIH (green) and HCs (red). LDA score on log_10_ scale indicated at bottom. Significance of the microbial marker increases with LDA score. AIH, autoimmune hepatitis; HCs, healthy controls; OTUs, operational taxonomic units; LEfSe, linear discriminant analysis effect size; LDA, linear discriminant analysis.

A LEfSe analysis (Figure 5A) and the LDA genus score (Figure 5B) confirmed that 27 microbial biomarkers in AIH clearly distinguished between patients with AIH and the HCs. Moreover, the divergence between both groups was highly significant (P < 0.05). Biomarker names, LDA scores, log values and *P*-value are shown in Supplementary Data S6.

Cladogram in Figure 5A using LEfSe method indicated that the phylogenetic distribution of intestinal microbiome associated with patients with AIH (green) and healthy controls (red). The AIH microbiome was characterized by a preponderance of *Lactobacillaceae, Family_XI*, Verrucomicrobiaceae, *Veillonellaceae, Enterobacteriaceae* and *Pasteurellaceae*, whereas the HCs microbiome was characterized by a preponderance of *Lachnospiraceae, Desulfovibrionaceae, Porphyromonadaceae*, and *Alcaligenaceae*.

LDA scores in Figure 5B showed the significant bacterial genus difference between AIH and HCs. Ten genera including *Veillonella, Faecalibacterium, Klebsiella, Gemella, Akkermansia*, and *Lactobacillus* predominated in AIH patients (*P* < 0.05; LDA > 2). Twenty genera including *Phascolarctobacterium, Incertae_Sedis, Sutterella, Ruminococcus, Pseudobutyrivibrio*, and *Lachnospira* predominated in the HCs (*P* < 0.05; LDA > 2).

### Gene Function Analysis

**Figure 6:**
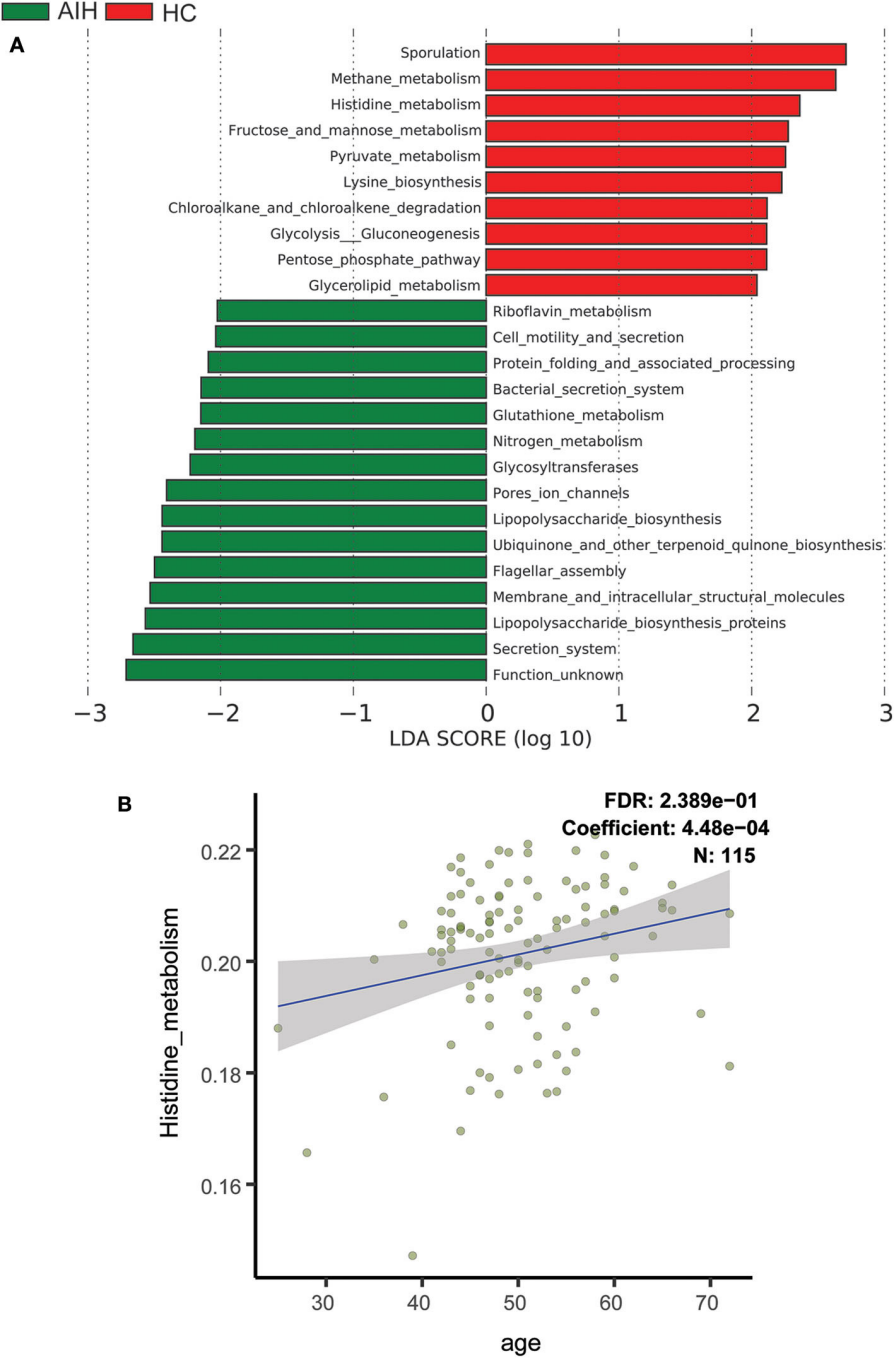
Functional analysis of predicted metagenomes. **(A)** Differentially abundant KEGG pathways across AIH (*N* = 37) and HCs (*N* = 78) identified by LEfSe. Histogram of LDA scores calculated for selected pathways showing significant difference in gene functions between AIH (green) and HCs (red). LDA score on log_10_ scale indicated at bottom. Significance of the microbial marker increases with LDA score. AIH, autoimmune hepatitis; HCs, healthy controls; OTUs, operational taxonomic units; LEfSe, linear discriminant analysis effect size; LDA, linear discriminant analysis. **(B)** Scatterplots of relative abundances of histidine metabolism and age. A positive correlation was found between the abundance of histidine metabolism and age (Spearman's correlation coefficient = 4.48e−04, FDR = 0.2389, N = 115). AIH, autoimmune hepatitis; LDA, linear discriminant analysis; LEfSe, linear discriminant analysis effect size; MaAsLin, multivariate association with linear models algorithm.

To elucidate the functional and metabolic alterations of the intestinal microbiomes between AIH and HCs group, the metagenomes were next inferred from the 16S rRNA data and the functional potential of the gut microbiota were analyzed using PICRUSt. The differentially abundant KEGG pathways across the AIH (*N* = 37) and HC (*N* = 78) groups were identified by LEfSe (Figure 6A). Twenty-four pathways were validated by multivariate association with linear models algorithm (MaAsLin), adjusting for covariates. Ten pathways including methane metabolism, chloroalkane and chloroalkene degradation, pyruvate metabolism, and lysine biosynthesis were enriched in the HCs (*P* < 0.05; LDA > 2). Fifteen pathways including bacterial secretion, glutathione metabolism, riboflavin metabolism, cell motility and secretion, nitrogen metabolism, lipopolysaccharide biosynthesis, and protein folding and associated processing were enriched in the AIH patients (*P* < 0.05; LDA > 2). Notably, glutathione metabolism was highly enriched in the AIH microbiome while pyruvate metabolism and lysine biosynthesis were significantly depleted in AIH, which might be associated with disease status.

Scatterplots Figure 6B demonstrated a positive correlation between abundance of histidine metabolism and age (Spearman's correlation coefficient = 4.48e−04; FDR = 0.2389; *N* = 115). Thus, the histidine metabolism pathway should be excluded from the analysis as it is highly correlated with age.

### Correlation Between Fecal Microbiome and AIH Disease Status

**Figure 7:**
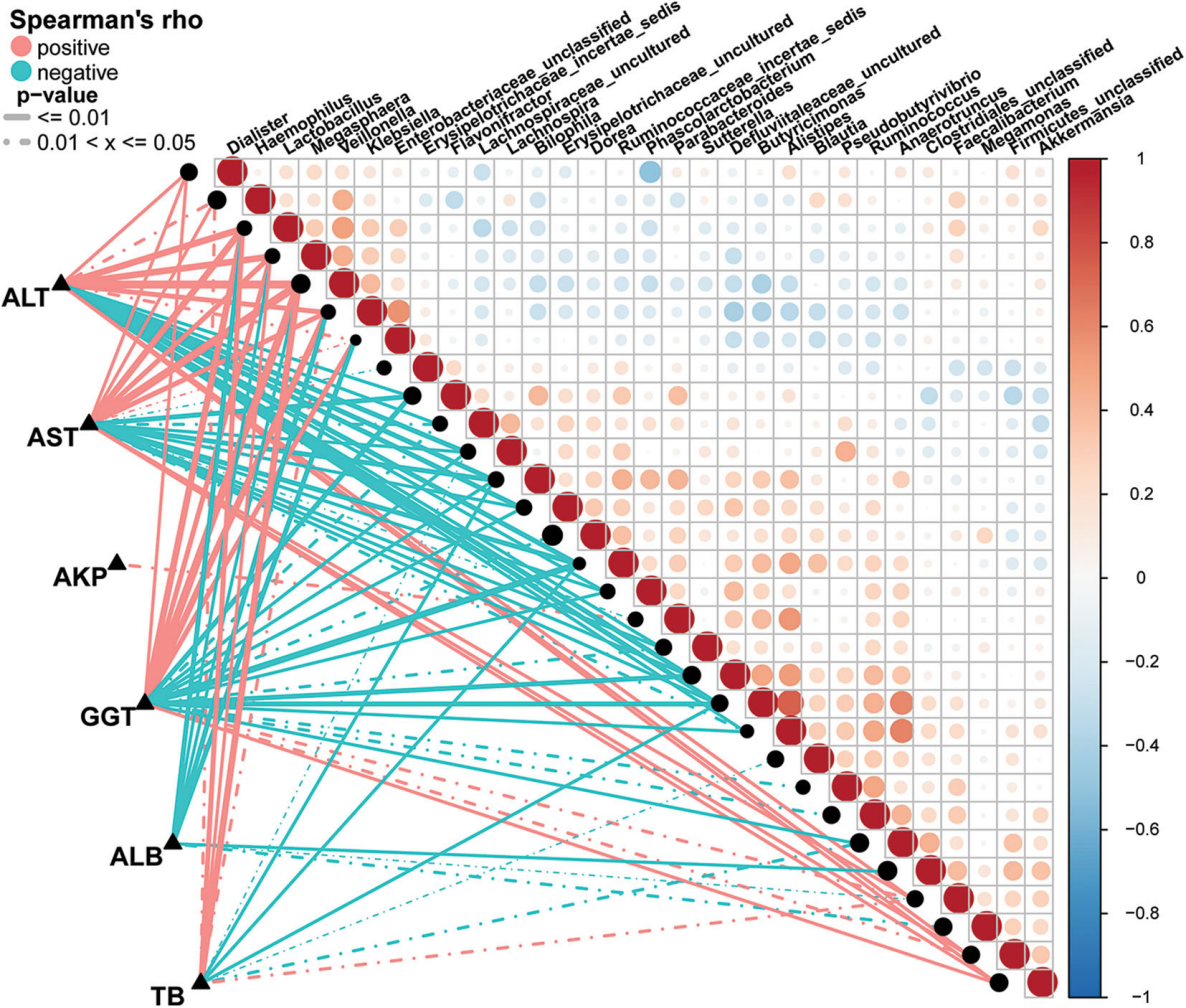
Associations between fecal microbiomes and AIH disease status. Heatmap showing partial Spearman's correlation coefficients among 30 genera and clinical AIH indices. Positive values (red) indicate positive correlations. Negative values (blue) indicate inverse correlations. Solid lines represent *P* ≤ 0.01. Dotted lines represent 0.01 < *P* ≤ 0.05. Intensity of shading in circles is proportional to the magnitude of the association. Correlation direction was determined by Spearman's method. *P < 0.05; **P < 0.01; ***P < 0.001; AIH, autoimmune hepatitis; AST, aspartate aminotransferase; ALT, alanine aminotransferase; GGT, γ-glutamyltransferase; TB, total bilirubin; ALB, albumin; AKP, alkaline phosphatase.

A Spearman's rank test was performed to analyze correlations between the AIH-associated genera and the clinical characteristics of individuals with AIH. Potential interferences such as age, gender and BMI were considered. The distance correlation plots in Figure 7 (Supplementary Data S7) revealed partial Spearman correlation coefficients among 30 genera and the clinical indices AST, ALT, GGT, TB, ALB, and AKP of the AIH group. There were positive correlations between AST and the abundances of ten genera including *Veillonella, Lactobacillus, Megasphaera, Klebsiella*, and *Akkermansia* (*P* < 0.05). Moreover, these ten aforementioned genera were positively correlated with ALT. However, 13 genera including *Ruminococcaceae_incertae sedis, Flavonifractor, Bilophila* and *Butyricimonas* were negatively correlated with AST (*P* < 0.05). There were significant positive correlations between the abundances of *Veillonella, Lactobacillus, Megasphaera, Klebsiella*, and *Faecalibacterium* and AST, ALT, GGT, and TB in the AIH group (*P* < 0.05). *Parabacteroides* was the only genus positively correlated with AKP (*P* < 0.05). Eight genera including *Lactobacillus, Klebsiella*, and *Enterobacteriaceae_unclassified* were negatively correlated with ALB. No genera were positively correlated with ALB.

### Potential Use of Fecal Microbiome-Based Signatures in AIH Diagnosis

**Figure 8:**
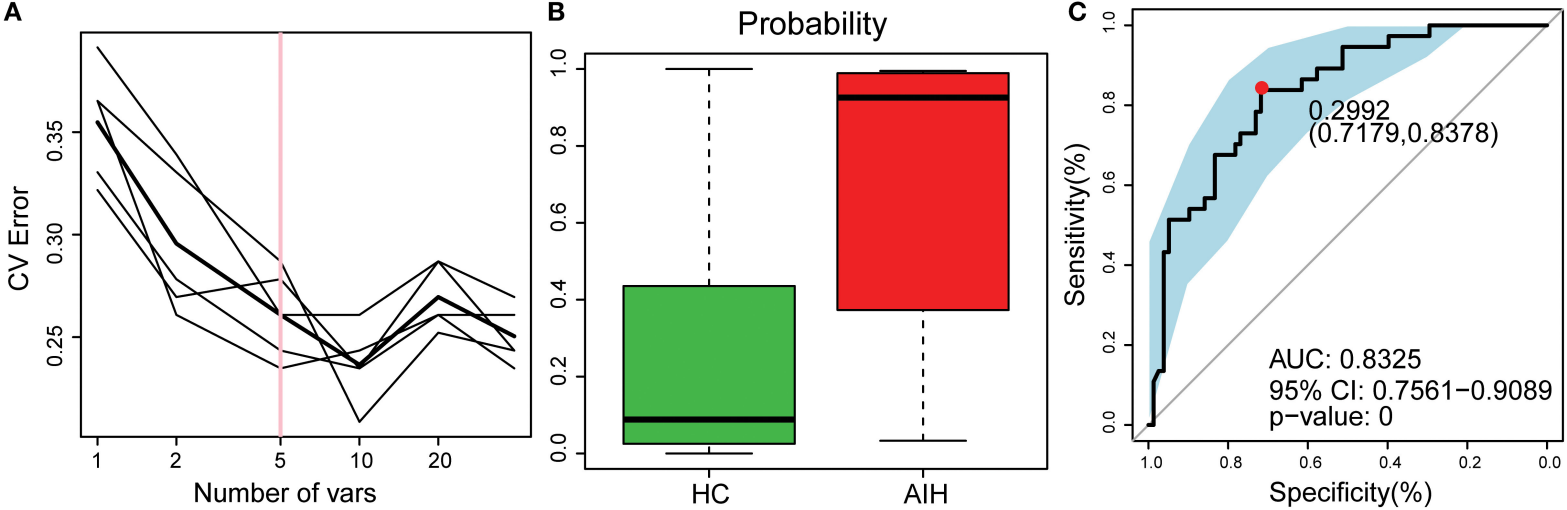
Identification of microbial OTU-based AIH markers by random forest models. **(A)** Five OTUs were selected by random forest models as the optimal AIH biomarkers. **(B)** POD was significantly higher in AIH than HCs. **(C)** The POD index had AUC = 83.25% with 95% CI = 75.61–90.89% between AIH and HC. AIH, autoimmune hepatitis; HCs, healthy controls; OTUs, operational taxonomic units; CV error, cross-validation error; POD, probability of disease; AUC, area under the curve.

The smallest OTU combination that could accurately identify the differences between the AIH and HCs groups was established. A cross-validation curve revealed five OTU biomarkers including *Lachnospiraceae, Veillonella, Bacteroides, Roseburia*, and *Ruminococcaceae* (Figure 8A).

**Figure 9:**
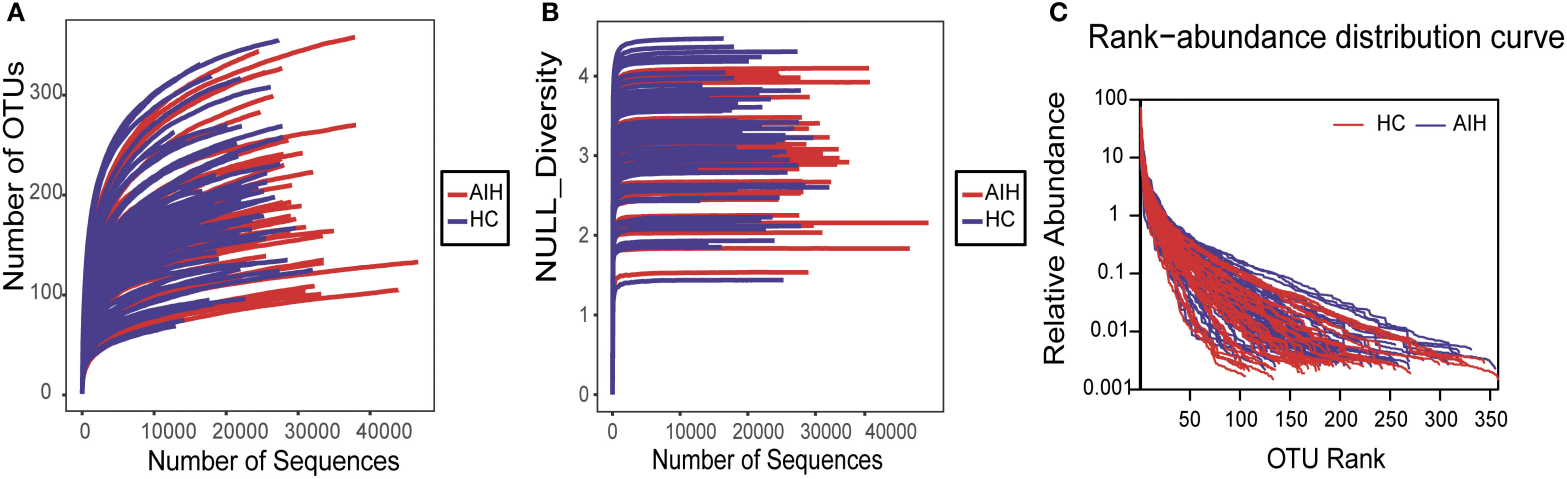
Data quality of bacterial 16S rRNA gene sequences. **(A)** Rarefaction analysis showed that estimated OTUs richness basically approached saturation in each group, and it was significantly increased in AIH (*n* = 37) (red) vs. HCs (*n* = 78) (blue). Similar results were also obtained in **(B)** Shannon-Wiener curve and **(C)** Rank-Abundance curve, showing that the sequencing data of the sample is of high quality. AIH, Autoimmune hepatitis; HCs, healthy controls; OTUs, Operational Taxonomy Units.

**Figure 10:**
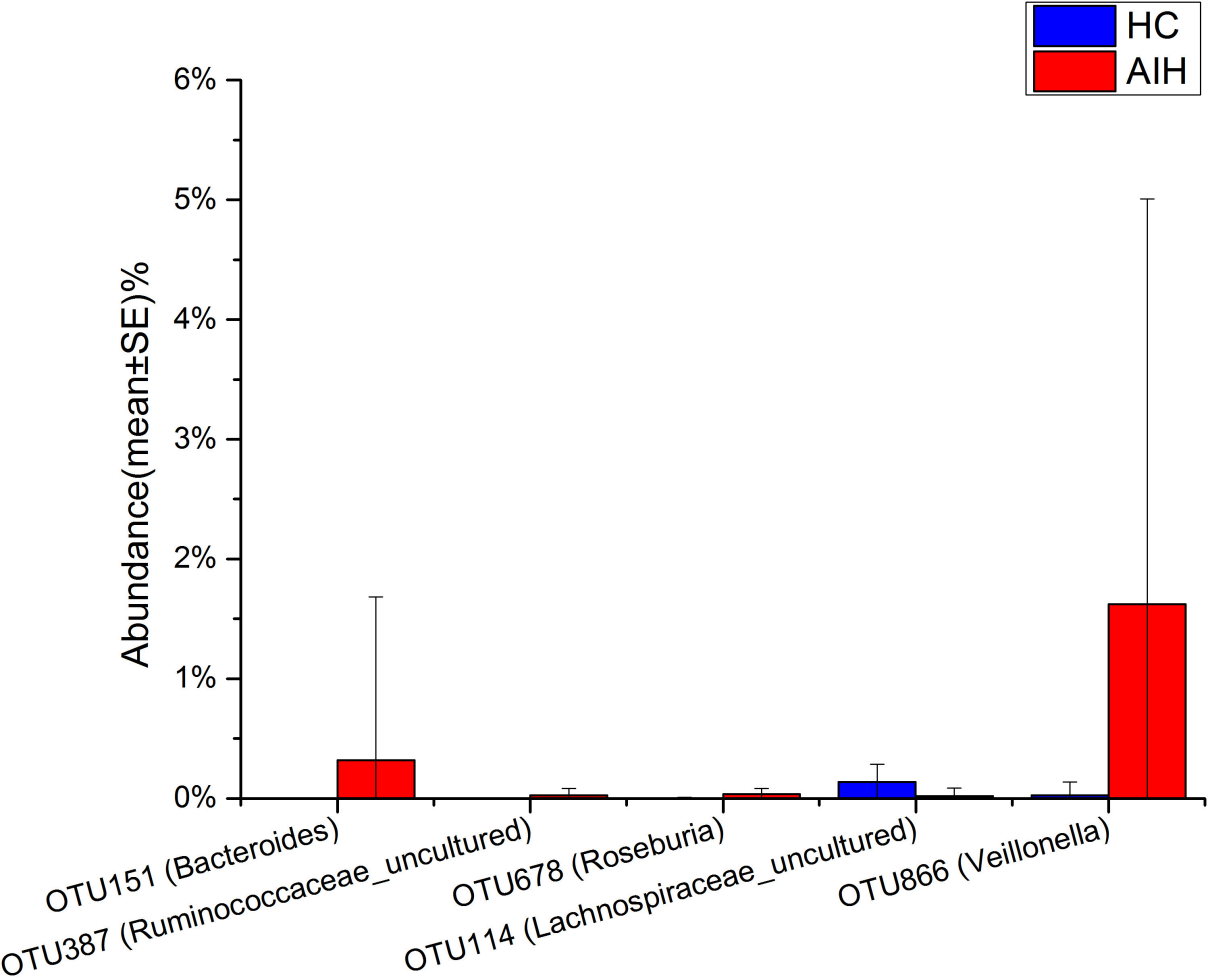
Relative abundances of five OTUs between AIH (*N* = 37) and HCs (*N* = 78). The relative abundance of five OTUs between AIH (blue) and HCs (red) is shown in histogram. The relative abundance of each OTU is depicted as mean ± SE. *P* were calculated by a Wilcoxon rank-sum test. *P < 0.05; **P < 0.01; ***P < 0.001; AIH, autoimmune hepatitis; HCs, healthy controls; OTUs, operational taxonomic units.

**Figure 11:**
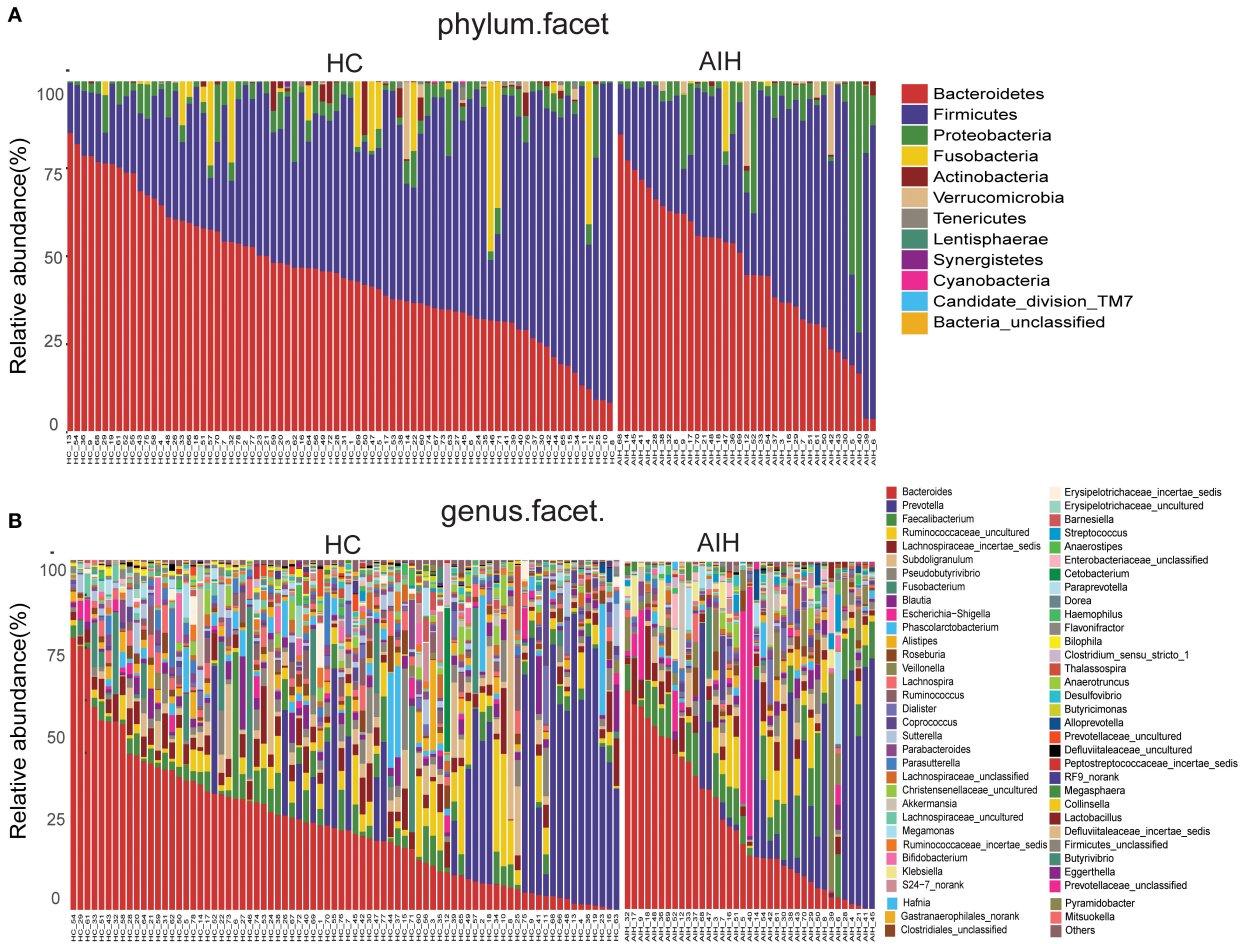
Composition of fecal microbiota on taxonomic level between AIH (*n* = 37) (right) versus HCs (*n* = 78) (left). The microbial community barplot of AIH and HCs visually reflected the relative abundance of microbiome at **(A)** phylum level and **(B)** genus level for each sample. AIH, Autoimmune hepatitis; HCs, healthy controls.

**Figure 12:**
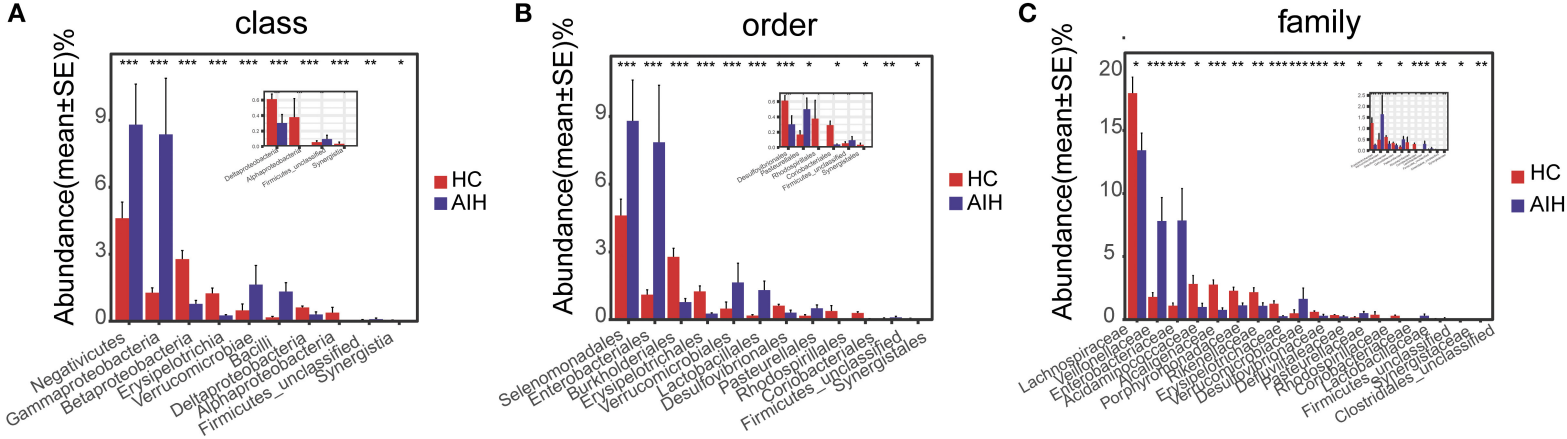
Comparison of fecal microbiome on taxonomic level between AIH (*N* = 37) and HCs (*N* = 78). Comparison of fecal microbiota at **(A)** the class level; **(B)** order level and **(C)** family level between AIH (blue) and HCs (red). The average abundance values for each bacterium is depicted as mean ± SE. *P* values were calculated using the Wilcoxon rank sum test, and are shown in Supplementary Data S3-Data S5. Significant differences by *P < 0.05; **P < 0.01, and ***P < 0.001. AIH, Autoimmune hepatitis; HC, healthy controls.

The microflora data and the five OTU biomarkers were used to calculate the POD index (Figure 8B). It was markedly higher in the AIH group than that in HCs group.

The AIH-associated microbial genera distinguish AIH from HCs. The AUC was ~0.8325 and the 95% confidence interval was 0.7561–0.9089 (*P* < 0.001) (Figure 8C).

## Discussion

Previous studies reported the importance of fecal microbiomes in various liver disorders such as chronic hepatitis B (CHB) (Wang et al., 2017), cirrhosis (Qin et al., 2014), non-alcoholic fatty liver disease (NAFLD) (Caussy et al., 2019), HCC (Ren et al., 2019), and PBC (Tang et al., 2018). Notably, Wei Y et al demonstrated the alterations of gut microbiome in AIH patients in East China (Wei et al., 2019). However, it is still important to perform microbiome studies of AIH in different geographic populations.

In this study, we identified the differences in fecal microbiome between AIH patients and HCs in Central China for the first time, and indicated the efficacy of fecal microbiome as a diagnostic tool for AIH. A total of 37 patients with AIH and 78 matched HCs underwent 16S rRNA gene sequencing. The former exhibited distinct overall microbial compositions compared to the latter. Ten genera including *Veillonella, Faecalibacterium, Akkermansia*, and *Lactobacillus* were predominated in AIH, while twenty genera including *Sutterella, Ruminococcus, Pseudobutyrivibrio*, and *Lachnospira* were enriched in HCs. The analysis of NMDS and PCoA confirmed a significant divergence between the AIH and HCs in terms of fecal microbial community composition. Based on a random forest model, five OTUs including *Lachnospiraceae, Veillonella, Bacteroides, Roseburia*, and *Ruminococcaceae* were selected as the optimal biomarkers to distinguish AIH patients from HCs, and achieved an AUC value of 83.25%, suggesting that fecal microbiomes could distinguish the AIH patients from healthy individuals.

Most of the findings in this study corroborate previous reports with several exceptions. There are some differences and advantages of this paper from other published study. Our study is mainly aimed at the intestinal microbial difference of AIH patients in Central China, whereas the patients in the research of (Wei et al., 2019) are from East China. Our results showed that there were significant differences of five bacteria between AIH patients and healthy individuals, in which *Ruminococcaceae, Veillonella, Roseburia*, and *Bacteroides* were enriched in AIH group, while *Lachnospiraceae* was enriched in HCs group. The results in (Wei et al., 2019) showed that *Veillonella, Lactobacillus, Oscillospira*, and *Clostridiales* were significantly different in AIH and HCs group. Notably, the AUC value in our study achieved 83.25%, while the AUC value in Wei et al. (2019) was 78%.

The differences in the findings from the two studies are mainly attributed to different geographic population. Regional difference is the main influencing factor of intestinal microbial difference. A recent study of He et al. (2018) investigated the differences of intestinal flora in 10 000 people in different areas of Guangdong Province, and suggested that there was a significant relationship between intestinal microbial differences and regional differences. Accordingly, it is pivotal and necessary to study the differences of intestinal microbiome of AIH patients in different regions in order to elucidate the possible pathogenesis of AIH and to establish a diagnostic model. In this paper, we present the characteristics of AIH patients in Central China for the first time, and have certain innovation and reference value.

Moreover, in our study, the diagnostic criteria of AIH were based on the patients' liver function, the autoantibodies and the immunoglobulins, which was consistent with the diagnostic methods presented in the guidelines (Lohse et al., 2015). The subjects included in our study were out-patients with AIH, which were relative early stage, with only mild symptoms. They had never been treated with steroids and UDCA, since these drugs might greatly impact the microbiome analysis. Therefore, the microbial diagnosis model established by us realizes the early diagnosis and non-invasive diagnosis of AIH patients. From the view point of clinical diagnosis and treatment, our study has a significant clinical application value.

Furthermore, we found the reduction of *Ruberia* and *Ruminoccocaceae* in the AIH patients. So we speculate that the alterations of intestinal microbiomes may be involved in the occurrence and development of AIH. The components of intestinal microorganisms or their metabolites promote the progression of AIH. Targeted intestinal microbiota may be a new therapeutic target for AIH. *Ruberia* and *Ruminoccocaceae* are protective bacteria, which provide a clear target for the treatment of AIH. Increasing these two bacteria may prevent the occurrence and development of AIH.

*Ruminococcus* degrades mucin, produces short chain fatty acids (SCFAs), and is diminished in certain autoimmune disorders such as IBD, AIH, and psoriatic arthritis. SCFAs may inhibit effector T cell activation and repress redundant local and systemic inflammatory responses (Makki et al., 2018). *Ruminococcus* may promote SCFA production, increase the number of Th1 and Th17 cells, and upregulate IL-10 which is anti-inflammatory and crucial to intestinal homeostasis. When *Ruminococcus* abundance is substantially decreased, metabolic inflammation may be induced (Liao et al., 2019).

As a new diagnostic tool for diseases, gut microbiome has attracted more, and more attention. The gut microbiome have been reported to involve in various diseases like cirrhosis (Qin et al., 2014), NAFLD (Caussy et al., 2019), HCC (Ren et al., 2019), and PBC (Tang et al., 2018). These microbial markers have many advantages when applied to disease diagnosis, such as high accuracy and efficiency, especially their non-invasive nature.

For instance, Shen et al. (2017) evaluated the relationship between gut dysbiosis and NAFLD. NAFLD patients had lower gut microbiota diversity than healthy subjects. Proteobacteria and Fusobacteria phyla were more abundant in NAFLD patients. Additionally, Loomba et al. (2017) recently reported an American cohort that included patients with NAFLD and fibrosis. They identified a set of 40 features, which included 37 bacterial species that were used to construct a random forest classifier model to distinguish mild/moderate NAFLD from advanced fibrosis with a high AUC value of 0.936. These findings represent a major advancement in the field of gut microbiome research, and specifically, its use as a diagnostic marker for disease.

The sample size of the present study was a little small, which is mainly attributed to the fact that the number of newly diagnosed patients with AIH is much smaller than that of revisit patients. Most of the clinical patients with AIH are revisit patients who have received drug treatment. However, the subjects included in our study were newly diagnosed patients with AIH, which had never been treated with steroids and UDCA. Due to the strict inclusion and exclusion criteria, the final sample size is a little small. Therefore, the establishment of non-invasive diagnostic model for AIH using gut microbiota requires larger clinical samples and multicenter clinical studies to validate.

Additionally, another serious disadvantage of this paper is that individual's microbiology was associated with many confounding factors such as time (the community types of stool sample were more unstable before freezing), diet, environment and antibiotic treatment. For example, in an 8-week in-hospital study, Ang et al. (2020) performed gene sequencing and metabolomics analysis of fecal samples and found significant changes in gut microbial community structure and function during Ketogenic Diets, which suggests that diet does interferes with intestinal microecology. And also, those functional aspects which are simply extrapolated from the compositional data cannot be guaranteed to be deterministic in practice. In order to minimize the interfering effects of confounding factors, the only thing we can do is to enlarge the sample size, manage to improve the standardization of sampling procedure to minimize the influence of other interfering factors. Accordingly, a much more systematic study would examine a large, randomly selected sample of individuals with various diet habits, gender, age and from different areas.

In conclusion, this study was the first to present the characteristics of gut microbiomes in AIH patients in Central China. The microbial diagnosis model was established to distinguish patients with AIH from healthy individuals in Central China, realizing the early diagnosis and non-invasive diagnosis of AIH patients.

## Data Availability Statement

The datasets generated for this study can be found in the European Nucleotide Archive Database of the European Bioinformatics Institute, accession number PRJNA556801.

## Ethics Statement

The study was approved by the Institutional Review Board of the First Affiliated Hospital of Zhengzhou University (2017-XY-002). The patients/participants provided their written informed consent to participate in this study.

## Author Contributions

ZY and ZR designed the study. JL, YJ, BR, SD, HY, HZ, ZL, and QS collected clinical samples and performed the experiments. AL and GC analyzed the data. JL and ZR wrote the manuscript. All authors reviewed and approved the manuscript.

## Conflict of Interest

The authors declare that the research was conducted in the absence of any commercial or financial relationships that could be construed as a potential conflict of interest.
