# Supplementary material for: Fecal Microbiomes Distinguish Patients With Autoimmune Hepatitis From Healthy Individuals
Source: Front Cell Infect Microbiol. 2020 Aug 3;10:342. doi: 10.3389/fcimb.2020.00342 (PMC7416601; doi:10.3389/fcimb.2020.00342)
Supplement: Supplementary file 1 [file Table_1.docx]

| Table 1 Clinical characteristics | | | | |  |
| --- | --- | --- | --- | --- | --- |
| Characteristics | **AIH(n=37)** | **HC(n=78)** | **P value** | | |
| Age, years,  median(min–max) | 50  (25-72) | 49.5  (36-65) | | 0.469 | |
| Gender, Female, n(%) | 34(91.89%) | 67(85.9%) | | 0.358 | |
| BMI, kg/m2,  median(min–max) | 21.89  (18.47-29.34) | 22.04  (18.31-29.34) | | 0.962 | |
| Hepatic function, median (min–max) | | | | | |
| ALT, U/L | 160(47-553) | 16(7-38) | | 0.000- | |
| AST, U/L | 95(40-407) | 20(11-33) | | 0.000 | |
| AKP, U/L | 70(40-263) | 69(32-157) | | 0.296 | |
| GGT, U/L | 90(54-321) | 17(7-49) | | 0.000 | |
| TB, umol/L | 20(6-71) | 11.305(3.8-18.12) | | 0.000 | |
| ALB, g/L | 36(29-54) | 47.35(39.6-53.2) | | 0.000 | |
| Immunoglobulin, median (min–max) | | | | | |
| IgG, g/L | 21.3(9,82-44) |  | |  | |
| IgM, g/L | 1.5(0.51-4.2) |  | |  | |
| IgA, g/L | 1.8(0.6-8.63) |  | |  | |
| Autoantibody, +/-, +% |  |  | |  | |
| ANA | 33/4; 89.19% |  | |  | |
| ASMA | 5/32; 13.51% |  | |  | |
| SLA/LP | 9/28; 24.32% |  | |  | |

Continuous variables were compared using Wilcoxon rank sum test between both groups. Fisher’s exact test compared categorical variables. Statistical analyses were performed using SPSS version 20.0 for Windows (SPSS Inc., Chicago, IL).

BMI, body mass index; AIH, autoimmune hepatitis; ALT, alanine aminotransferase; AST, aspartate aminotransferase; AKP, alkaline phosphatase; GGT, gamma-glutamyltransferase; TB, total bilirubin ; ALB, albumin
