# Supplementary material for: Fecal Microbiomes Distinguish Patients With Autoimmune Hepatitis From Healthy Individuals
Source: Front Cell Infect Microbiol. 2020 Aug 3;10:342. doi: 10.3389/fcimb.2020.00342 (PMC7416601; doi:10.3389/fcimb.2020.00342)
Supplement: Supplementary file 2 [file Data_Sheet_1.docx]

**Supplementary** **Information**

**Title: Fecal microbiome distinguish patients with** **autoimmune hepatitis from healthy individuals**

**Running title: Fecal microbiome of patients with autoimmune hepatitis**

Authors: Jiamin Lou 1,4†, Yan Jiang 2†, Benchen Rao 1,4, Ang Li 1,4, Suying Ding 3, Hang Yan 3, Heqi Zhou 1,4, Zhenguo Liu 1,4, Qingmiao Shi 1,4, Guangying Cui 1,4, Zhigang Ren 1,4*, Zujiang Yu 1,4*

City: Zheng*zhou, China*

**Affiliations**

^1^ Department of Infectious Diseases, the First Affiliated Hospital of Zhengzhou University, Zhengzhou 450052, China

^2^ Department of Neurology, the First Affiliated Hospital of Zhengzhou University, Zhengzhou 450052, China

^3^ Physical Examination Center, the First Affiliated Hospital of Zhengzhou University, Zhengzhou 450052, China

^4^ Gene Hospital of Henan Province; Precision Medicine Center, the First Affiliated Hospital of Zhengzhou University, Zhengzhou 450052, China

***Correspondence:**

Zhigang Ren, Ph.D., M.D., Department of Infectious Diseases, the First Affiliated Hospital of Zhengzhou University, 1#, Jianshe East Road, Zhengzhou 450052, China. E-mail: [fccrenzg@zzu.edu.cn](mailto:fccrenzg@zzu.edu.cn)

Zujiang Yu, Ph.D., M.D., Department of Infectious Diseases, the First Affiliated Hospital of Zhengzhou University, No. 1, Jianshe East Road, Zhengzhou 450052, China. E-mail: [johnyuem@zzu.edu.cn](mailto:johnyuem@zzu.edu.cn)

^†^These authors contributed equally to this work.

**Supplementary Information**

1. **Informed consent form and information collection in the study (Translated from Chinese) 3-4**
2. **Informed consent form for scientific research 5-7**

**3. Supplementary figures S1-4:**

**Supplementary figure 1 8**

**Supplementary figure 2 9**

**Supplementary figure 3 10**

**Supplementary figure 4 11**

**Informed consent form and information collection**

**(Translated from Chinese)**

We are from Gene Hospital of Henan Province; Precision Medicine Center; Department of Infectious Diseases, the First Affiliated Hospital of Zhengzhou University. We will free of charge help you monitor your fecal microbial community, thereby analyzing whether fecal microbiota is dysbiosis and the degree of imbalance. These results will provide auxiliary data for clinical diagnosis and treatment. Now, you just provide stool according to our instruction. The whole process keeps free of charge. These results will be used for scientific research. Thank you for your corporation.

Number:

Diagnosis:

Patient Sign：

Date:

**Patient information collection**

| Name | Gender | Birth date | Height (cm) | Weight (kg) | BMI | Tel |
| --- | --- | --- | --- | --- | --- | --- |
|  |  |  |  |  |  |  |
| Floor  ward | Admission number | Dietary habit (vegetarian diet/meat/Mixture) | Antibiotics use within 2 months | Yoghourt and probiotics | Previously critical Illness | Long-term drug use |
|  |  |  |  |  |  |  |
| Drinking | Alcohol  type | Drinking quantity | Time of Duration | Whether or  not abstinence | HBV | Etiology |
|  |  |  |  |  |  |  |

**Informed consent form for scientific research**

**(Translated from Chinese)**

Dear participants,

We are from Gene Hospital of Henan Province; Precision Medicine Center; Department of Infectious Diseases, the First Affiliated Hospital of Zhengzhou University. We will free of charge help you monitor your healthy condition and record your clinical information and healthy/disease status or disease progression process. The collected fecal samples from participants in hospital will be used for scientific research. These results and data from the hospital electronic medical records will provide auxiliary data for clinical diagnosis and treatment, and will be used for scientific research. Thank you for your corporation.

Number: Diagnosis:

The information that we collect from this research project will be kept confidential. Information about you that will be collected during the research will be put away and no-one but the researchers will be able to see it. Any information about you will have a number on it instead of your name. Only the researchers will know what your number is and we will lock that information up with a lock and key. It will not be shared with or given to anyone except our research team.

The knowledge that we get from doing this research will be shared with you through community meetings before it is made widely available to the public. Confidential information will not be shared. There will be small meetings in the community and these will be announced. After these meetings, we will publish the results in order that other interested people may learn from our research.

I have read the foregoing information, or it has been read to me. I have had the opportunity to ask questions about it and any questions that I have asked have been answered to my satisfaction. I consent voluntarily to participate as a participant in this research.

Print Name of Participant__________________

Signature of Participant ___________________

Date ___________________________

Day/month/year

A literate witness must sign (if possible, this person should be selected by the participant and should have no connection to the research team). Participants who are illiterate should include their thumb-print as well.

I have witnessed the accurate reading of the consent form to the potential participant, and the individual has had the opportunity to ask questions. I confirm that the individual has given consent freely.

Print name of witness_____________________ AND Thumb print of participant

Signature of witness ______________________

Date ________________________

Day/month/year

Statement by the researcher/person taking consent

I have accurately read out the information sheet to the potential participant, and to the best of my ability made sure that the participant understands that the following will be done:

1. We will free of charge help you monitor your healthy condition and record your clinical information and healthy/disease status or disease progression process.

2. These data from hospital electronic medical records will be used for scientific research.

3. The collected fecal samples will be used for scientific research.

I confirm that the participant was given an opportunity to ask questions about the study, and all the questions asked by the participant have been answered correctly and to the best of my ability. I confirm that the individual has not been coerced into giving consent, and the consent has been given freely and voluntarily.

 A copy of this ICF has been provided to the participant.

Print Name of Researcher/person taking the consent________________________

Signature of Researcher /person taking the consent__________________________

Date ___________________________

Day/month/year

**Supplementary Figure 1**

**
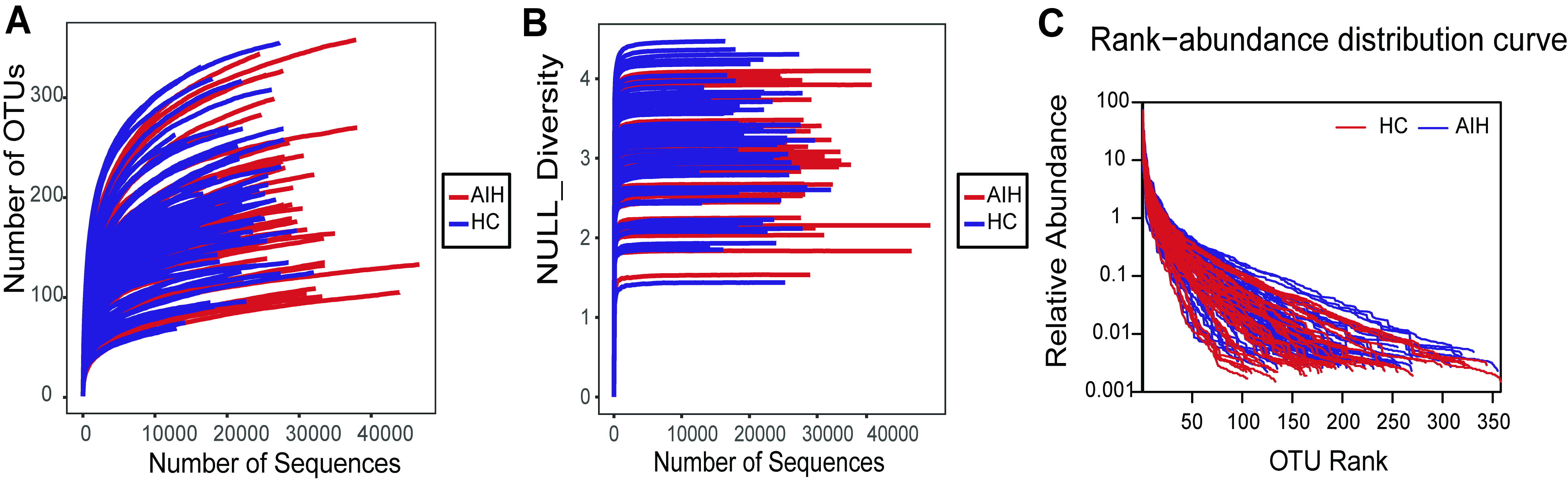
**

**Figure S1. Data quality of bacterial 16S rRNA gene sequences.** **(A)** Rarefaction analysis showed that estimated OTUs richness basically approached saturation in each group, and it was significantly increased in AIH (n=37) (red) versus HCs (n=78) (blue). Similar results were also obtained in **(B)** Shannon-Wiener curve and **(C)** Rank-Abundance curve, showing that the sequencing data of the sample is of high quality. AIH, Autoimmune hepatitis; HCs, healthy controls; OTUs, Operational Taxonomy Units.

**Supplementary Figure 2**


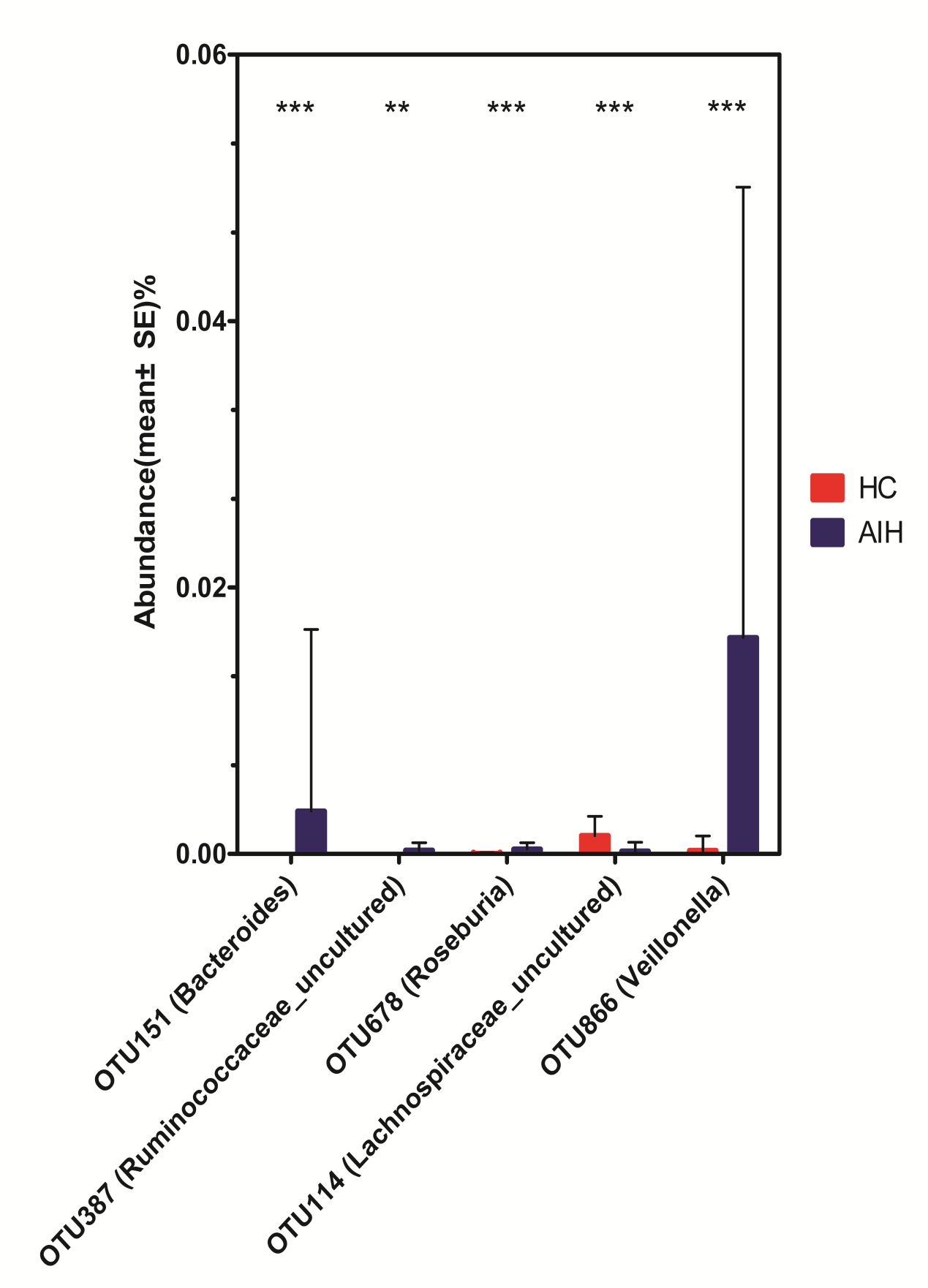


**Figure.S2** **Relative abundances of five OTUs between AIH (N = 37) and HCs (N = 78).** The relative abundance of five OTUs between AIH (blue) and HCs (red) is shown in histogram. The relative abundance of each OTU is depicted as mean ± SE. P were calculated by a Wilcoxon rank-sum test. *P < 0.05; **P <0.01; ***P < 0.001; AIH, autoimmune hepatitis; HCs, healthy controls; OTUs, operational taxonomic units.

**Supplementary Figure 3**

**
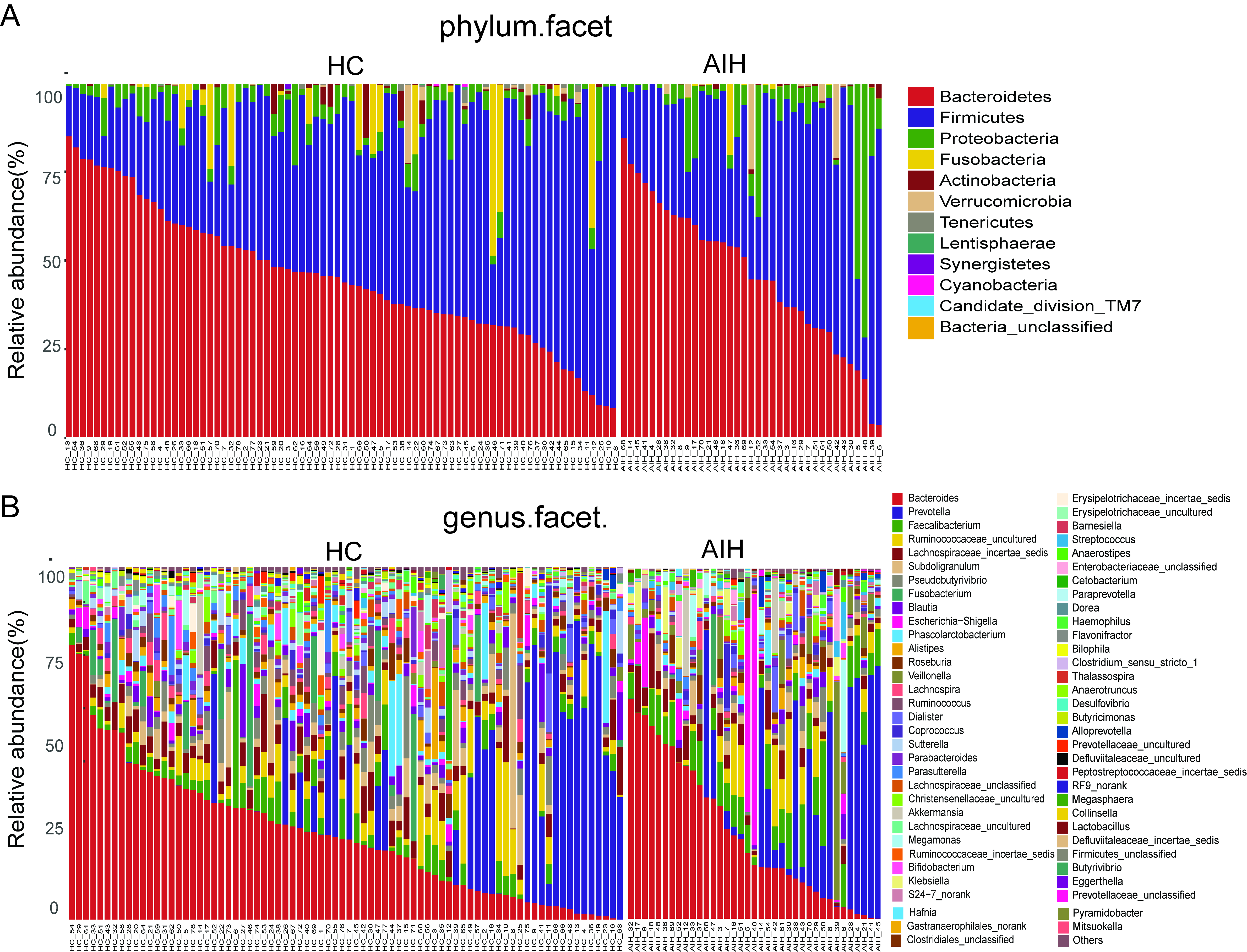
**

**Figure S3. Composition of fecal microbiota** **on taxonomic level between AIH (n=37) (right) versus HCs (n=78) (left).** The microbial community barplot of AIH and HCs visually reflected the relative abundance of microbiome at **(A)** phylum level and **(B)** genus level for each sample. AIH, Autoimmune hepatitis; HCs, healthy controls

**Supplementary Figure 4**

**
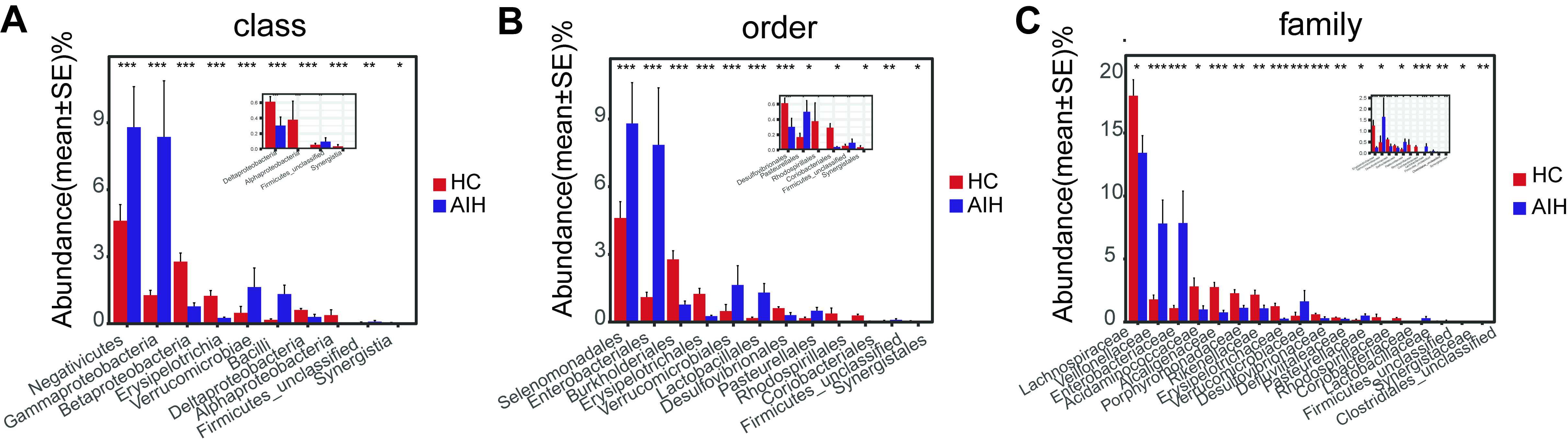
**

**Figure S4.** **Comparison of fecal microbiome on taxonomic level between AIH (N=37) and HCs (N=78).**  Comparison of fecal microbiota at **(A)** the class level**; (B)** order level and **(C)** family level between AIH (blue) and HCs (red). The average abundance values for each bacterium is depicted as mean ±SE. P values were calculated using the Wilcoxon rank sum test, and are shown in supplementary Data S3; Data S4 and Data S5. Significant differences by *P < 0.05; **P <0.01 and ***P < 0.001.AIH, Autoimmune hepatitis; HC, healthy controls.
